# Modern Technologies in Diagnosis of Fungal Keratitis (Review)

**DOI:** 10.17691/stm2023.15.2.07

**Published:** 2023-03-29

**Authors:** A.V. Sitnova, S.N. Svetozarskiy

**Affiliations:** 6-year Student, Medical Faculty; Privolzhsky Research Medical University, 10/1 Minin and Pozharsky Square, Nizhny Novgorod, 603005, Russia;; Ophthalmologist; Privolzhsky District Medical Center of the Federal Medico-Biological Agency (FMBA), 14 Ilyinskaya St., Nizhny Novgorod, 603000, Russia Tutor, Department of Eye Diseases; Privolzhsky Research Medical University, 10/1 Minin and Pozharsky Square, Nizhny Novgorod, 603005, Russia;

**Keywords:** corneal mycosis, keratitis diagnosis, pathogenesis of infectious keratitis, soft contact lenses, confocal microscopy, OCT, PCR diagnosis

## Abstract

Traumas and infectious diseases of the eye play a leading role in the development of corneal blindness responsible for 1.5–2 million cases of vision loss per year. To date, the issue of reducing the incidence of fungal keratitis is acute and needs to be solved worldwide. Trauma as a risk factor for corneal fungal disease is thought to be prevalent in developing countries due to agricultural involvement, while in developed countries the onset of the disease is predisposed by medical advances such as contact vision correction and modern ophthalmic surgery. Thorough analysis of the pathogenesis gives the possibility to describe the action of fungal enzymes, biofilm formation, and the resistance mechanism, which on the one hand explains the aggressive course of the disease and difficulties in its diagnosis, and on the other hand, it encourages searching for new methods of diagnosis and treatment. The non-specific clinical picture of fungal keratitis, the variety and availability of antibiotics nowadays become an obstacle for rapid detection of this pathology. Low public awareness and late visit to an ophthalmologist are also a barrier to successful combating the increasing incidence of fungal keratitis. Belated diagnosis, increasing resistance of fungi to antibiotics, and lack of registered antifungal ophthalmic drugs justify poor treatment efficacy resulting in decreased visual acuity or vision loss**.**

Existing diagnostic methods need systematization and detailed comparison, identifying the advantages and disadvantages of each. This review considers causative agents and their influence on pathogenesis of the disease, describes difficulties of fungal keratitis diagnosis and possible ways of overcoming these problems using new developments, and also outlines further prospects of research in this direction.

## Introduction

Fungal keratitis is an infectious disease characterized by the affection of the cornea by pathogenic fungi, severe course, and a high risk of unfavourable outcome with loss of vision and an eye as an organ.

According to some microbiologists, medical mycology has for a long time played the role of Cinderella in the microbiological family [1, 2]. In recent years, with the widespread and often irrational use of antibiotics and increased number of patients receiving hormonal and immunosuppressive therapy, fungal infections have become much more frequent, and medical, social, and economic damage related to them is of great concern [3, 4]. The same trend is also observed in relation to the cases of mycotic keratitis, the share of which among the general number of infectious keratitis has been gradually growing for the last decades [5, 6]. Thus, in 2017–2019, the corneal mycosis morbidity did not fall below 1 million cases per year [7, 8]. Annually, 1.5–2 million people experience sight loss caused by corneal pathology [9]. The majority of cases occur in developing countries with hot and humid climate where a substantial part of the population is involved in agriculture. Thus, from 1999 to 2020, Paraguay, Ethiopia, Sri Lanka, Bangladesh, India, and China were the leading countries in the incidence of fungal keratitis [10]. Besides, ophthalmic operations and soft contact lenses (SCL) are considered to be risk factors. If one of these events causes infection, a chain of the processes leading to a deeper injury of the cornea is triggered. Pathogenesis of mycotic keratitis includes corneal damage directly by a fungal agent and indirectly by the rapidly developing inflammatory reaction.

Prevalence of the disease does not always provide diagnostic accuracy, especially in the regions with low alertness of doctors. Clinicians from the USA have established that American ophthalmologists defined correctly infectious keratitis in 73% of cases among patients with positive inoculation results, a while fungal etiology was identified only in 38% of cases [11]. Inapparent clinical picture and absence of pathognomic signs limit the possibility of early identification and timely administered specific treatment.

Diversity of fungal infection, diagnostic difficulties, lack of officinal ophthalmic preparations, universal therapeutic schemes, and ways of preparation delivery explain both frequent need of surgical intervention and high risks of reduction and vision loss: every year, 84,143– 115,697 eyes are removed due to the complications of fungal keratitis [8]. According to the literature data, corneal perforation caused by fungal infection develops 6 times more often than in keratitis of other etiology [12, 13], keratoplasty is required more frequently [14]. To avoid these unfavorable consequences, the search for a new optimal diagnostic method are constantly being carried out, since inoculation of fungal culture and direct microscopy of the stained smears, being the golden standard, do not meet all current requirements. This method must be simple enough and experience-independent, affordable, must fast and accurately define the etiological factor. Such method combined with the analysis for sensitivity to antibiotics will allow ophthalmologists to choose further tactics and improve prognosis.

### Strategy of literature search

PubMed (MEDLINE) and eLIBRARY.RU databases were searched for published works using the following key words: “keratomycosis or fungal keratitis”; “fungal diseases”, “eye”. The list of references in the appropriate articles helped find thematic publications.

## Etiology

The fungal kingdom includes over 1.5 million species, and only some of them are known to be pathogenic to humans. In recent years, classification of fungi previously based on morphological features has undergone considerable changes connected with the fact that now genotypic differences constitute its basis. As a result, significant regrouping of species among the known fungi has taken place. Clinically important is the division of the pathogenic fungi into filamentous and yeast morphologically and into zoophilous, geophilous, and antropophilous by their natural habitat. The latter live in the human body and many of them are able to cause keratitis. The most common and therefore most significant pathogenic fungi for clinicians are worth considering. The analysis of clinical cases of fungal keratitis over the last 20 years has shown that *Fusarium* genus was found in 40% of cases, *Aspergillus* in 31%, *Curvularia* in 6%, while the yeast fungus of the *Candida* genus became the cause of the disease in 4.5% of cases (of them, *C. albicans* made up 67.87%) [10]. The spectrum and proportion of the etiological factors vary from region to region even within the limits of one country. In some studies, the incidence of the *Fusarium* genus reached 61.9% [15], *Candida* — 72.22% [16]. The complaints of patients are similar: sensation of the foreign body, lacrimation, photophobia, eye reddening, blurred and reduced vision [17]. None of the symptoms is pathognomic for fungal keratitis, and it is often impossible to differentiate it from the infections of other etiology due to the similar clinical picture [18]. Difficulty in identifying the etiological factor and a wide diversity of causative agents prevent accurate diagnosis and selection of etiotropic therapy, therefore specialists have to rely on the comprehensive history-taking and characteristic risk factors revealed.

## Risk factors

Healthy cornea is considered resistant to infection by fungal agents, and therefore there must be factors predisposing to contamination and causing infection to develop [19].

Risk factors for fungal keratitis are as follows: eye traumas — from 26% [20] to 39% [21] of cases; surgical interventions on cornea — 37.6% [22]; wearing contact lenses — from 24.5% [23] to 42% [20]; corneal diseases — from 28% [23] to 51.3% [22]; systemic and local immunosuppression — 35.9% [22].

As a rule, fungal infection is preceded by mechanical damage of the cornea, and therefore the most common risk factors are traumas and interventions breaking its integrity. The most dangerous are traumas caused by the objects of organic and inorganic nature (branches, leaves, stones, sand) containing soil particles. Contact correction is sometimes accompanied by microtraumas, especially when instructions for SCL are not observed; constant lens wearing can also cause local hypoxia and hypercapnia which influence the ability of epithelium to respond to damage [24]. The number of registered Fusarium keratitis in patients using SCL is growing [25, 26], and rare cases of keratitis induced by opportunistic *Arthrograthis kalrae* are in absolute majority connected with contact correction [27]. According to the results of one of the investigations [22], application of SCL was more often associated with keratitis caused by filamentous fungi (50%) than by yeast ones (18.2%), while ailments of the ocular surface were a widespread risk factor at any etiology. Of all ophthalmic operations, penetrated keratoplasty creates the greatest danger for fungal keratitis emergence [28], besides fungal keratitis may recur in the graft [29]. It is interesting that different variants of layer-by-layer keratoplasty have a high risk of mycotic complications (0.023% [30]) in comparison with penetrated keratoplasty (from 0.012 [30] to 0.016% [31]). It has been noted that fungal keratitis complicates regeneration after transplantation of endothelium and Descemet membrane not only in the early postoperative period (0.15% of cases [32]) but several years after the operation [32, 33]. Cases of complications after implantation of Boston type 1 keratoprosthesis (kpro) have been described in the literature [34].

It should be noted that even more sparing interventions do not guarantee absence of complications. Soleimani and Haydar [35] reported the case of severe unilateral fungal keratitis four days after minimally-invasive SMILE laser vision correction. The generated ulcer did not respond to medicamentous treatment and required penetrated keratoplasty due to perforation. Because of the intrastromal lenticule location and consequently fast spreading of the infection into the deep corneal layers, treatment of infectious keratitis after SMILE turned out to be very difficult [35].

A serious risk factor of developing fungal corneal damage is the application of local antibiotics and corticosteroids [36]. Inhibiting the inflammatory process, corticosteroids aggravate immunosuppressive effect caused by fungi. At the beginning, a period of apparent improvement from corticosteroids is possible, but soon the condition becomes worse: corneal infiltration grows, the amount of secretion increases, vision reduces [37]. Besides, in some cases, the application of corticosteroids after the operation for fungal keratitis may result in reinfection [35].

Depending on the type of the infectious agent and the way of its penetration into the corneal depth, the pathogenesis of fungal keratitis may have some specific features, but in any case it is a complex process influenced by many factors.

## Pathogenesis

The struggle between a fungal microorganism and the immune system of a macroorganism is often fierce enough, since factors of fungal pathogenicity are diverse and the immune response is not always adequate. Microorganism pathogenicity and a biological burden are the leading factors determining the severity of keratitis course [38].

Fungi enter the corneal depth through the epithelial defect and release mycotoxins, proteases, and lectines. Immune cells identify the foreign agent with the receptors of pattern recognition and then neutrophils, macrophages, and dendritic cells start to actively secrete chemokines (CXCL1 and CXCL2) and proinflammatory cytokines (IL-1b and TNF), which attract more and more immune cells to the cornea to eliminate the pathogen [39]. An excessive amount of neutrophils and growing inflammation cause keratitis progression resulting in stroma damage and corneal opacity [40, 41], forming a vicious circle where inflammation becomes the reason of further tissue injury and vision loss.

### Factors of fungal pathogenicity

Mycotoxines secreted by fungi possess antibacterial, antiviral, antitumor, and antiphagocytic effect, inhibit a local immune response. A lot of fungi secrete proteases: for example, *Candida spp*. produce acidic, neutral, and carboxyl proteases, which increase the invasive ability of a fungus [42]. Lectines in their turn suppress the growth of the corneal cells and destroy the cellular structure of the epithelium [43].

Hydrophobin is an insoluble protein complex on the spore surface which together with mycotoxins makes phagocytosis difficult since hydrophobin prevents recognition of fungi by the immune cells [44].

It should be separately noted that many fungal species are able to form biofilms [45] which are presently distinguished as a separate pathogenetic factor [46]. The biofilms represent a constantly renewing community of microorganisms secured on the substrate and surrounded by a polymer matrix which protects them against detrimental effects [47– 49]. Thus, *Fusarium* fungi often form biofilms on the SCL surface [50], and this determines the prevalence of Fusarium keratitis in contact correction. The biofilm structure of that kind defends against environmental factors, increases pathogen resistance to the immune defense and antifungal agents, facilitates adhesion, invasion, and spread of the infection in the host tissues [46]. Using *C. albicans* as an example, it has been shown that biofilm forms inhibit the release of neutrophil extracellular traps and resist neutrophil attack [51]. The studies have proved that biofilms increase minimum inhibitory concentration (MIC) of antifungal medications (sometimes MIC exceeds those for plankton forms by 100 times and more) and play the role in resistance formation [45, 52]. Antimycotics capable of affecting the biofilms are few and all of them induce formation of reactive oxygen species (ROS) [50].

Probably, it is the ability of fungi to suppress the immune response that explains why in some observations the amount of inflammatory cells in the cornea is in reverse proportion to the fungal content, and in the process of fungus growth, the inflammatory process weakens [17, 53]. Together with these significant specific features of pathogenesis, application of local corticosteroids results in the protracted course and addition of secondary infection in case of surgical treatment [35].

### Factors of microorganism defense

The increased expression of pattern recognition receptors in response to a foreign agent leads to the secretion of interleukins IL-1b, IL-6, IL-8, IL-17, and IL-23 by neutrophils [54]. IL-1b promotes ROS formation inhibiting the growth of fungal hyphae. At the same time, ROS induces more intensive production of IL-1b and may damage the surrounding tissues. It has been noted that some fungi start to synthesize antioxidants under the oxidative stress and overcome this protective mechanism [55]. As a consequence, the existing inflammation becomes more intensive [56].

It has been proved that contamination by some fungi, for example, *C. albicans* and *Aspergillus spp.*, triggers the process of autophagy representing degradation of organelles and proteins in eukaryotic cells and being a regulator of intracellular homeostasis [57, 58]. Autophagy decreases chemotaxis of neutrophils and the damaging action of the pathogen, promotes elimination of intracellular pathogens and weakens the inflammatory reaction in general, and in this connection this process is considered to play a key role in the immune response [57].

### Specific features of pathogenesis and its dependence on etiology

The genus *Fusarium* as a representative of filamentous fungi is characterized by “horizontal” growth of hyphae parallel to collagen fibers causing damage to the superficial corneal layers, whereas “vertical” growth normally to collagen fibers is more typical to the genus *Aspergillus* and *Candida* yeasts disrupting the normal arrangement of collagen fibers and permitting the causative agent to penetrate into the deep stromal layers [59, 60]. It is specific to *Candida* to produce phospholipase A, facilitating penetration to the tissues, and lysophospholipase protecting the yeast cells against the action of other enzymes [61]. Filamentous fungi proliferate in the corneal stroma not releasing any chemotaxic substances, which again delays the development of the immune response. In case of the disease progression, fungi pass through the previously intact Descemet membrane [42, 62]. Moreover, fungal keratitis may occur secondary to fungal endophthalmitis. In this case, damage starts from the anterior segment, the pathogen overcomes the Descemet membrane and affects the corneal stroma [63]. Thus, the “horizontal” growth and protection from the immune response in keratitis caused by filamentous fungi, are associated with a less favorable outcome and consequently with the need of surgical treatment [64]. The investigations show that a single operative intervention may be insufficient when damage is caused by filamentous fungi. There have been described cases of treating corneal ulcer caused by *Aspergillus spp.*, when penetrated keratoplasty has to be repeated 4 times due to fungal infiltrates in keratograft or emergence of endophthalmitis [65].

Specific features of pathogenesis are a key for timely diagnosis and identification of a causative agent, promoting administration of adequate etiotropic therapy in the sufficient dosage.

## Diagnosis

Establishing the etiology of keratitis is the necessary condition for determining the tactics and prognosis of treatment, whereas species identification of a pathogen is of secondary significance. The investigation designed from these positions has demonstrated that practicing ophthalmologists succeed in the attempts to differentiate exactly fungal from bacterial keratitis using photographs only in 66% of cases [66]. Causes of belated diagnosis may be absence of pathognomic symptoms, often a sluggish course and inapparent clinical picture, ability to mimic keratitis of other etiology. Sometimes, fungal keratitis may be taken by a patient for konjunctivitis and is treated with antibiotics and anti-inflammatory medications in order to arrest the symptoms. Even in African countries with the leading incidence of mycotic corneal damages, patients often delay their visits to a specialist to about 14 days, and attendance of several medical settings increases the delay to about 21 days [67]. Situation like this decreases the chances for timely diagnosis and treatment. A case of severe torpid course of the disease was described in Kazakh Research Institute of Eye Diseases where a woman was admitted 4 months after the trauma caused by a cow tail. Despite a complex treatment, a threat of perforation remained for a month and ultimately there was formed a total opacity of the cornea [68]. These examples show how important it is to increase awareness of patients and their trust in doctors.

Doctors’ alertness, their experience, and well-equipped clinics play a leading role in early identification of fungal keratitis. Works on creation and testing various diagnostic methods speak of the importance of the problem. Urgency of the issue in the tropical countries brought about the creation of an express-method using a folding microscope based on a smartphone as an alternative to a light microscope in the regions with limited resources [69].

### Biomicroscopy

There are several signs which may help suspect fungal corneal damage using the biomicroscopy technique [70-72]:

cloud-like or caseous, multifocal, grayish infiltrates with feathery or scalloped borders;

satellite infiltrates located near the main focus and separated from it by a clear area [42];

ring-shaped infiltrates;

mycelioid stromal overgrowths;

endothelial plaques (are not visualized in marked corneal infiltration) [73].

Apart from the manifestations typical for the majority of fungal keratitis, there may be indented or “feathery”, indistinct margins in Fusarium ulcers and an elevated surface of the foci in Aspergillus keratitis, which is more often accompanied by hypopyon [74]. In case of yeast-like fungi-induced stromal keratitis, a small protruding ball-shaped infiltrate can be seen [75]. None of the signs is pathognomic for fungal keratitis, for example, ring-shaped infiltrates are also characteristic for acanthamoeba keratitis [76-78]. Today, cultural method and direct microscopy of corneal scrapings with staining remain the leading diagnostic methods.

### Cultural method

The biological material is used for inoculation of the growth medium to cultivate microorganisms and evaluate the colony obtained. The material for culturing may be collected by corneal scraping, biopsy, or penetrated keratoplasty. Then, the specimen is seeded on the growth medium, for example on the liquid glucose peptone Sabouraud culture medium, blood and chocolate agars, or brain heart infusion agar [79]. Genus and sometimes species of the causative agent are identified by specific appearance of the colony (color, shape, consistency, spore availability, hypha, pseudohypha). This method is considered the golden standard of the diagnosis, is easy enough, and cost-effective, but still having some limitations: difficulty of species diagnosis, long response time (about a week) [80, 81], dependence on the researcher experience, non-typical morphology of some colonies, insufficient knowledge of the suitable conditions of cultivation, inaccessibility of stromal layers for corneal scraping in case of deep fungal penetration [82], false-negative results of inoculation in case of insufficient scraping volume or progression of corneal destruction, the effect of the previous empirical therapy on the inoculation results [83]. For example, one of the studies [84] reported negative results of inoculation and unestablished laboratory diagnoses in 37% of patients with fungal keratitis explaining the reason by antibiotic therapy administered before the admission to the hospital. It is worth mentioning that in case of positive culture detection, identification of a causative agent is successful only in 40–60% of cases [17].

At present, it is recommended to repeat inoculation six days after the beginning of etiotropic therapy in order to assess the efficacy of treatment and clarify the prognosis [85]. It has been found that in case of the positive repeated seeding, the risk of perforation and the necessity of penetrated keratoplasty increases. Thus, inoculation six days after the start of treatment may help correct therapy, avoid critical reduction of vision acuity and operative intervention [86].

### Direct microscopy with staining

This method is fast and easy to perform. Different ways of specimen staining are usually used: Gram or Giemsa staining, fixation with potassium hydroxide solution, staining with lactophenol cotton blue, Shiff reagent, Gomori methenamine silver staining [85, 87]. Corneal scrapes, bioptate, or a corneal fragment taken during penetrated keratoplasty may serve as a biomaterial.

Staining in direct microscopy allows for visualization of hyphae and their relative position, evaluation of mycelium type [87]. Sensitivity of the method varies depending on the staining technique and reaches on average 90% [85, 88]. Being cheap, simple, and fast, the microscopy makes it possible to administer promptly etiotropic therapy [85]. Along with the cultural method, microscopy is a basic stage in the diagnosis, although it has also some drawbacks. The successful application of this method depends on the depth of corneal damage, amount and quality of the collected material, and researcher experience. The probability of non-uniform staining of the preparation and artifact detection is also high; besides, this method is not always successful in relation to the *Candida* genus [85, 87].

### Confocal microscopy

Confocal microscopy provides *in vivo* imaging of the yeast and mold fungi in the corneal tissue. This method has its advantages owing to non-invasiveness and ability to overcome restrictions of classical techniques, its sensitivity is in the range of 66.7‒95.0% [89]. The convincing criteria of corneal damage by mycelial fungi are clearly outlined, branching, strongly reflecting filaments or hyphae of 3–10 μm in diameter usually not observed separately. In case of mold fungi, pseudohyphae representing dotted structures with discontinuities or constrictions are visualized [89]. Confocal microscopy enables determination of hyphae density while assessment in dynamics helps predict treatment response, since in the course of successful therapy the density of hyphae decreases [90]. This method gives a faster and, in some studies, a more accurate result than smear microscopy and inoculation. Jin et al. [73] have established that fungal keratitis diagnosed by confocal microscopy is successful in 92.9% of cases, by smear examination only in 71.4%, and by culturing in 42.9% of cases. The results of confocal microscopy are validated by the microscopy of the stained corneal tissue. In some cases, culturing and polymerase chain reaction (PCR) concede confocal microscopy meaning that the technique may be used for an early express-diagnosis [91]. At the same time, Ren et al. [83] detected fungal pathogens using both confocal microscopy and smear investigation in 77.14% of cases, which points to limitations of this diagnostic method. Inability to identify a causative agent species, high cost, insufficient experience of ophthalmologists carrying out examinations [92, 93], and a small specimen size may be referred as the limiting factors [94]. The procedure is strongly hindered by photofobia and blepharospasm [95].

### Optical coherence tomography (OCT)

Using this method, it is possible to detect changes in the cornea typical for the mycotic process. The OCT data show thickening of the cornea in the infiltrate region, hyperreflectivity of the epithelium and endothelium compared to the stroma. The stroma diffusely thickens showing evidence of edema which in its turn results in the alteration of the posterior corneal surface. In the prolonged course, scarring processes develop enhancing stroma reflectivity; in this case, the affected stroma may become thinner than the healthy areas [20]. Limited cystic formations of different sizes in the stroma corresponding to necrotized tissues are specific OCT signs of aggressive forms of fungal keratitis [96].

Using OCT and confocal microscopy, one can visualize over 85% endothelial plaques typical for fungal keratitis [73] which are almost indistinguishable during slit-lamp examination, especially in the presence of corneal edema and infiltration [6]. This method is fast, non-invasive, and more widely used than confocal microscopy. The signs of corneal damage are only indirect evidence of the fungal pathogen presence and, therefore, make species identification impossible, but the OCT technique is convenient for evaluation of the corneal state in dynamics, and enables to trace changes over the entire cornea.

### Polymerase chain reaction

The PCR diagnosis pertains to the methods of molecular genetic diagnosis and is not inferior to or even exceeds microscopy of the stained preparations and cultural methods detecting fragments of fungal DNA even in cases with negative culturing results [81, 97].

The advantages of PCR are indisputable: the results may be obtained within 4 h instead of 3–7 days in cultural investigations; the method is highly sensible enabling to detect a pathogen in a small scrape from the corneal ulcer or the material from patients receiving previously antifungal therapy [81]. Like other molecular genetic methods, PCR is designed for species identification. However, it is considered as a method of choice due to its limited availability and high cost [98]; moreover, there is a high likelihood of false-positive results due to the fact that PCR identifies non-viable organisms as well [79].

### Other molecular genetic methods

Much more progressive and accurate method is a metagenomics analysis with the predominant evaluation of RNA and identification of the specimen species composition. Shigeyasu et al. [99] have presented the case when corneal specimens showed negative results in microbiological and histological investigations, and only metagenomics analysis succeeded in detecting the genes of *Fusarium solani*. In the other study [100], microscopy of the stained preparations has shown sensitivity of 70%, culturing was positive in 52% of patients, whereas metagenomics analysis has established the presence of a pathogen in 74% of cases, taking into consideration the fact that more than half of the patients have already undergone treatment. This technique supersedes the classical ones even in abundant contamination of the specimen [100].

Mass spectrometry, based on the analysis of microorganism ribosomal proteins, is also referred to the molecular genetic methods. Laser-assisted desorption/ionization time-of-flight mass spectrometry (MALDI-TOF MS) allows for identification of an agent up to a species level during 24 h, which is an undeniable advantage [21]. A tear from an affected and healthy eye is analyzed for its protein composition since in fungal lesions, the content of proteins changes in a specific way [96]. The investigations of MALDI-TOF MS sensitivity show contradictive results varying from 51% [21] to 97% [87]. Not readily available, expensive, requiring constant replenishment of the data bank with microorganism proteomes are the disadvantages that prevent this method from becoming the main technique in the diagnosis of fungal keratitis [101].

Ren et al. [83] have presented the first results of using a new method of sequencing by internal transcribed spacer (ITS). The ITS is a non-coding DNA sequence separating the repeated rRNA genes. This method is not restricted by a medium, time, fungal activity, and specimen size, and provides more complete information about the eye microbiome. The ITS has demonstrated the result comparable with the classical methods and confocal microscopy. Mean indicators of efficiency in combination with some drawbacks of ITS (dependence on the database integrity, the necessity to use several primers due to their strict specificity for separate types of fungi) allows one to conclude that this method seems to be an option to identification of pathogens in the diagnosis of fungal keratitis [83].

Despite a wide spectrum of the known diagnostic technologies, none of the methods meet all the requirements of clinical practice (see the Table), therefore the development of new approaches to the detection of fungal keratitis is going on [87]. In the *in vivo* investigations on the murine model of Aspergillus keratitis, a non-invasive probe consisting of fluorophore-labeled antifungal preparation has been tested. Caspofungin (CSF), an antibiotic of the echinocandin class, is aimed at the fungus-specific enzyme β-1,3-D-glucan synthase responsible for the biosynthesis of fungal cellular wall. The drug is used in subtherapeutic doses for local application not producing measurable systemic concentrations. DDAO 7-amino-9H-(1,3-dichloro-9,9-dimethylacridin-2-one) was used as a label. Thus, the cells bound to the (L-CSF–DDAO) probe fluoresce and may be visualized in the near-infrared region. According to the *in vitro* study, L-CSF– DDAO identified readily the elements of *Aspergillus spp.* hyphae, whereas separately CSF and DDAO did not induce any visible fluorescence. The *in vivo* analysis confirmed the ability of the probe to bind specifically to the fungal cells in the infected cornea [103]. Caspofungin is available in the form of solution for infusions and is used to treat mycotic infections (including keratitis) caused by *Candida spp.* and *Aspergillus spp.*, especially those resistant to other pharmaceuticals. Efficacy in relation to *Fusarium spp*. is limited, therefore, there is a need to find a new diagnostic method in case of Fusarium keratitis.

**Table:** The main characteristics of the diagnostic methods

Diagnostic method	Physical aspects of the method	Sensitivity	Advantages	Drawbacks
Biomicroscopy	Examination of the anterior eye segment at multiple magnification	Depends on ophthalmologist’s experience and clinical manifestations of fungal keratitis	Available, fast, simple, inexpensive, non-invasive	Oriented only to the ophthalmologist’s knowledge and experience, insufficiently effective due to the absence of pathognomic signs of fungal keratitis
Cultural method	Culturing of microorganisms on a special growth medium for pathogen identification by colony morphology	About 60% [91, 100, 102]	Available, simple, inexpensive	Long response time, the result depends on researcher’s experience, quality and site of specimen collection, and previous drug therapy
Direct microscopy with staining	Staining of corneal scraping or specimen for visualization of structural fungal components using light microscope	Up to 90%	Available, fast, simple, inexpensive	The result depends on researcher’s experience, quality and site of specimen collection
Confocal microscopy	Light microscopy option with greater resolution and possibility to obtain corneal image at various depths	Up to 95%	Fast, simple, non-invasive, convenient for evaluation of process dynamics	Not readily available, expensive. The result depends on researcher’s experience. Unable to identify agent species
Optical coherence tomography	Visualizes all eye structures due to analysis of intensity and time delay of the light reflected from them	Up to 85%	Fast, simple, non-invasive, suitable for evaluation of process dynamics	Limited availability. Insufficient specificity, unable to identify agent species
Polymerase chain reaction	Molecular genetic method able to find a DNA fragment of a specific pathogen in the specimen	Up to 94% [87]	Fast, able to identify agent species. A small corneal fragment is sufficient for examination	Limited availability, expensive. False-positive result is possible
Metagenomic analysis	Molecular genetic method able to find rRNA and/or DNA fragments of a specific pathogen	Up to 74%	Fast, identifies agent species	Almost unavailable, expensive
Mass spectrometry	Molecular genetic method aimed at determination of highly specific ribosomal proteins of the causative agent	Up to 97%	Fast, non-invasive, identifies agent species	Almost unavailable, expensive. Depends on completeness and integrity of the database
ITS sequencing	Molecular genetic method consisting in genome sequencing using internal transcribed spacer which separates repeated rRNA fragments	Up to 65% [83]	Fast, identifies agent species. A small corneal fragment is sufficient	Almost unavailable, expensive. Depends on completeness and integrity of the database

## Analysis of antimicrobial sensitivity

No matter how quickly and accurately was the causative agent identified, a specialist has often to encounter the obstacles in order to achieve favorable outcome while treating fungal keratitis, i.e. with the resistance of fungi to antimycotics. The resistance in the majority of cases is acquired, and currently it grows due to the uncontrolled use of antibiotics, administration of inadequate empiric therapy. Fungi developed a variety of adaptive mechanisms to resist the action of antifungal preparations. They may, for example, generate special transport systems in their cellular membranes to intensify the elimination of the preparation from the cytoplasm or change membrane configuration to prevent binding of the medication to it. To withstand the action of antimycotics of the polyene group interacting with the fungal ergosterol, mycelium synthesizes a cellular wall with a decreased ergosterol content [104].

These mechanisms either promote full resistance or result in the increase of MIC preparations, and ultimately the application of the previously effective antimycotic does not improve the condition while microorganisms continue enhancing their resistance. All this demonstrates the necessity of testing for sensitivity to antifungal preparations and determining MIC after the detection of fungal etiology. A number of clinical cases are described, in which such analysis was of critical significance since timely replacement of the ineffective antimycotic is a key to successful prevention of corneal perforation [105]. These observations remind of the importance of cultural methods for the treatment of fungal keratitis and of the risks related with administration of empirical therapy. Moreover, the fact of the growing resistance speaks of the necessity to develop non-drug ways of treatment as an alternative to pharmacotherapy.

## Conclusion

The existing methods of diagnosing fungal keratitis allow ophthalmologists to detect what is invisible during a routine examination. And frequently, the cultural method and direct microscopy with staining as the golden standard appear to be insufficient for timely identification of fungal keratitis, which is associated with specific pathogenesis in each case. The growing morbidity, medical, social, and economic significance become an impetus to search for new diagnostic techniques. Defining the requirements, uncovering benefits and drawbacks of each approach help investigators understand the main characteristics that a new technique must possess in order to become the golden standard. In recent years, the PCR diagnostic methods have been increasingly used and in some fields of medicine they became a routine practice. We consider the PCR diagnosis to be one of the promising methods of revealing fungal keratitis since the results may be obtained within 4 h, sensitivity of the method reaches 94%, and species identification of a pathogenic fungus may also be implemented. However, along with the current diagnostic techniques, the cultural method and direct microscopy remain relevant owing to their high availability and simplicity.
